# Predictors of the Dose-Effect Relationship regarding Unilateral Inferior Rectus Muscle Recession in Patients with Thyroid Eye Disease

**DOI:** 10.1155/2015/703671

**Published:** 2015-06-28

**Authors:** Yasuhiro Takahashi, Hirohiko Kakizaki

**Affiliations:** Department of Ophthalmology, Aichi Medical University, Aichi 480-1195, Japan

## Abstract

*Purpose*. To evaluate whether inferior rectus muscle (IRM) thickness, the degree of adipose change in the IRM, smoking status, and the previous history of orbital radiotherapy can predict the dose-effect relationship regarding unilateral IRM recession in thyroid eye disease (TED). *Methods*. Twenty-five patients were retrospectively reviewed. We calculated the largest IRM cross-sectional area and evaluated the degree of adipose change in the IRM using magnetic resonance imaging. The degree of adipose change and smoking status were classified using grading scales (0–3); previous orbital radiotherapy was graded as 0 when a history was not available and 1 when it was available. The correlation between the dose-effect relationship and the hypothesized predictive factors was evaluated using stepwise multiple regression analysis. *Results*. The multiple regression model, with the exception of the history of the previous orbital radiotherapy, estimated a significant dose-effect relationship for the parameters evaluated (*Y*_DOSE-EFFECT_ = 0.013*X*_IRM AREA_  − 0.222*X*_ADIPOSE_  − 0.102*X*_SMOKING_ + 1.694; *r* = 0.668; adjusted *r* ^2^ = 0.367; *P* = 0.005). *Conclusions*. The dose-effect relationship regarding unilateral IRM recession in TED could be predicted using IRM thickness, degree of intramuscular adipose change, and smoking status but could not be predicted using the previous orbital radiotherapy history.

## 1. Introduction

Inferior rectus myopathy is a common but severe sequela in patients with thyroid eye disease (TED) [1–3]. TED-related inferior rectus myositis induces fibroadipose changes, which cause inextensibility of the inferior rectus muscle (IRM), resulting in restrictive hypotropia [3–6]. Although mild hypotropia can be compensated for by a chin-up, severe hypotropia cannot be adjusted using any ocular or head positions. This causes impairment of single vision, which has a strong negative impact on the activities of daily living [3, 4].

Recession of inextensible IRM is the first step in the correction of a restrictive hypotropia in TED. For preoperative evaluation of the amount of recession, ophthalmologists use a common dose-effect relationship for IRM recession; namely, approximately 2 degrees of ocular deviation is modified by 1 mm of IRM recession [7, 8]. However, as interindividual variation in fibrous changes in the IRM results in very limited reproducibility, reoperation is frequently required [3, 6, 9]. Although the severity of fibrous changes cannot be directly examined, its evaluation from variation in IRM thickness [10] and adipose changes in the IRM on magnetic resonance imaging (MRI) may be helpful for precise preoperative evaluation of the dose-effect relationship.

Understanding other predictive factors for the dose-effect relationship may also ensure a more tailored IRM recession for TED patients. Smoking and orbital radiotherapy are representative factors involved in the aggravation of strabismus in TED patients. Smoking increases thyroid autoantibodies, causes severe orbital inflammation by inducing hypoxia, and decreases the response to medical treatment [5]. Orbital radiotherapy is used for reducing orbital inflammation in TED patients but occasionally leads to excessive orbital fibrosis [11]. However, the correlation between the dose-effect relationship and these factors has not been elucidated.

In the present study, we examined whether IRM thickness, the degree of adipose changes in the IRM, smoking status, and a previous history of orbital radiotherapy are predictors of the dose-effect relationship regarding unilateral IRM recession in TED patients.

## 2. Materials and Methods

This study is a retrospective chart review of all TED patients who underwent unilateral IRM recession, which was performed by one of the authors (Hirohiko Kakizaki) between March 2011 and August 2014. The study was approved by the Institutional Review Board (IRB) of Aichi Medical University and followed the tenets of the Declaration of Helsinki. We obtained informed consent from the patients for the IRM recession surgery. However, the IRB granted a waiver of informed consent from the patients for this study, based on the ethical guidelines for epidemiological research established by the Japanese Ministry of Education, Culture, Sports, Science and Technology and Ministry of Health, Labour and Welfare because this study was a retrospective chart review, not an interventional study, and because it was difficult to get consent from all of the patients studied several years prior. However, the IRB requested us to present an outline of this study to the public via the Website of Aichi Medical University, to provide an opportunity for patients to refuse participation in this study. Patient records were anonymized and were deidentified prior to analysis.

Patients with a history of orbital decompression surgery or prior strabismus surgery, with missing clinical data, and with a follow-up time of <3 months were excluded from the study. All patients included in the study were euthyroid and in the static or chronic “burnout” phase; they did not have inflammation in the extraocular muscles (EOMs) on MRI [1].

### 2.1. Data Collection

The following data were collected: age, sex, surgical side, MRI findings, smoking status, past history of orbital radiotherapy, amount of IRM recession, and preoperative and postoperative angles of ocular deviation.

### 2.2. MRI Examination

MRI was performed by means of a 1.5-Tesla scanner (MAGNETOM Avanto, Siemens Healthcare, Erlangen, Germany) using a head-neck surface coil with the patient located in the supine position. Coronal T1- (repetition time, 500 ms; echo time, 10 ms; field of view, 140 × 140 mm; matrix, 256 × 220; and section thickness, 3 mm with 0.6 mm of an interslice gap) and T2-weighted gradient-echo sequences (repetition time, 4000 ms; echo time, 100 ms; field of view, 140 × 140 mm; matrix, 256 × 220; and section thickness, 3 mm with 0.6 mm of an interslice gap) were acquired.

**Figure 1:**
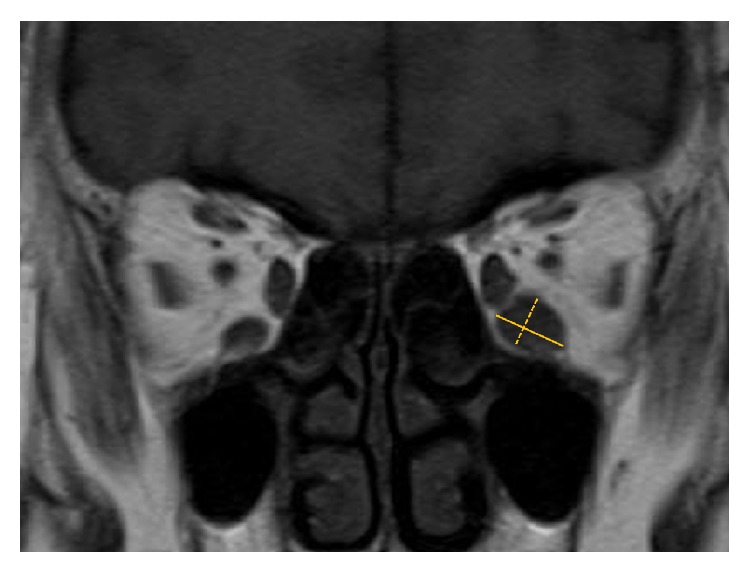
Measurement of the thickness of the inferior rectus muscle (IRM). The major axis of the IRM (solid line) and the minor axis perpendicularly crossing the major axis on the surgical side (dotted line) are measured on the T1-weighted coronal magnetic resonance image showing the largest IRM cross-sectional area.

IRM thickness was measured by one of the authors (Yasuhiro Takahashi) using the digital caliper tool of a viewer (ShadeQuest/ViewR, Yokogawa Medical Solutions Corporation, Tokyo, Japan). The major axis of the IRM on the surgical side was measured first on the T1-weighted MR image showing the largest IRM cross-sectional area (Figure 1). Next, the minor axis perpendicularly crossing the major axis was measured on the same MR image. We calculated the area of the largest IRM section, assumed to be an ellipse [12], as follows: (major axis/2) × (minor axis/2) × 3.14.

Adipose changes in the IRM were judged by one of the authors (Yasuhiro Takahashi) as high-intensity areas in the IRM muscle on both T1- and T2-weighted MR images. Every three consecutive T1- and T2-weighted images of the main portion of the IRM were used for evaluation. We classified the degree of adipose change in the IRM using an original grading system as follows: 0, no adipose change; 1, <1/4 of the IRM cross-sectional area; 2, 1/4–1/2 of the IRM cross-sectional area; and 3, >1/2 of the IRM cross-sectional area.

### 2.3. Smoking Status

Smoking status was classified by the number of cigarettes smoked per day, according to a report by Pfeilschifter and Ziegler [13] as follows: 0, no smoking; 1, <10 cigarettes/day; 2, 10–20 cigarettes/day; and 3, >20 cigarettes/day. All smokers were current smokers at the first examination, while all nonsmokers had not experienced smoking in their life.

### 2.4. Past History of Orbital Radiotherapy

The dose used for orbital radiotherapy was 20 Gy in all treated patients. The scale of previous orbital radiotherapy was expressed as 0 (without a history) and 1 (with a history).

### 2.5. Orthoptic Examination

The angle of ocular deviation was measured using a synoptophore at 1 day before surgery and 3 months after surgery. During the measurement, the fixing eye was set on the surgical side. The dose-effect relationship for IRM recession was calculated as follows: (preoperative angle − postoperative angle)/amount of IRM recession.

### 2.6. Surgical Technique

Surgery was performed under local anesthesia. A perilimbal conjunctival incision with radial relaxing incisions was undertaken in the inferior quadrant. A muscle hook was used to secure the IRM at its insertion, and Tenon's capsule around the IRM was thoroughly dissected using cotton swabs. The IRM tendon was secured using locking 8-0 polyglactin sutures (Vicryl, Johnson and Johnson Company, New Brunswick, NJ, USA) at 1 mm posterior to the globe insertion. Then, the IRM was detached from its insertion. The sutures were fixed to the sclera at 1 mm posterior to the point estimated from the preoperative ocular deviation angle. The recession of the IRM was calculated as follows: 2 degrees of ocular deviation per 1 mm IRM recession. The conjunctiva was closed using 8-0 polyglactin sutures.

### 2.7. Statistical Analysis

Patient age, the IRM cross-sectional area, and the dose-effect relationship were expressed as means ± standard deviations. The relationship between the dose-effect relationship and the hypothesized predictive factors was analyzed using stepwise multiple regression analysis. To examine whether smoking status and orbital irradiation were associated with the severity of intramuscular fibrous change, we compared the IRM cross-sectional area between smokers and nonsmokers and between patients with and without previous orbital irradiation using the Mann-Whitney *U* test. In addition, the proportion of patients with each degree of adipose change in the IRM was compared between smokers and nonsmokers and between patients with and without previous orbital radiotherapy using the chi-square test for independent variables. All statistical analyses were performed using SPSS Statistics version 22 software (IBM Japan, Tokyo, Japan). A *P* value of <0.05 was considered as being statistically significant in all tests.

## 3. Results

All of the data and the results from the statistical analyses are summarized in Tables 1–3. Twenty-five patients (9 men and 16 women; age, 61.3 ± 10.1 years; range, 38–78 years) were included. Surgery was performed on the right side in 13 patients and the left side in 12. Eight patients (6 men and 2 women) were smokers, and 12 patients (five men and seven women) underwent orbital radiotherapy.

A multiple regression model for the dose-effect relationship, the largest IRM cross-sectional area, the degree of adipose change in the IRM, and smoking status was created; however, previous orbital radiotherapy history was deleted from the model by the stepwise procedure. The model estimated a significant dose-effect relationship (*Y*_DOSE-EFFECT_ = 0.013*X*_IRM AREA_ − 0.222*X*_ADIPOSE_ − 0.102*X*_SMOKING_ + 1.694; *r* = 0.668; adjusted *r* ^2^ = 0.367; *P* = 0.005). A coefficient of “*X*” variable indicated a positive or negative correlation to the dose-effect relationship. The mean dose-effect relationship was 2.27 ± 0.6 degrees/mm.

**Figure 2:**
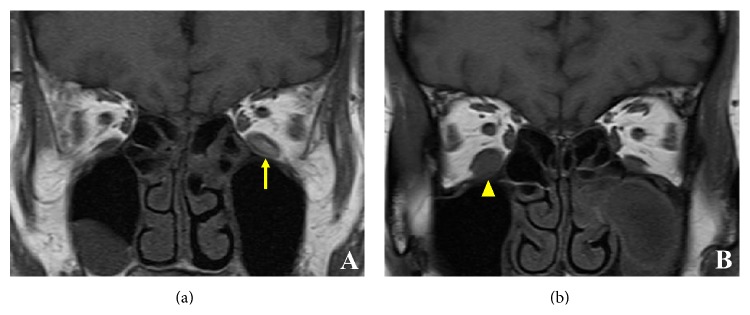
Adipose changes in the inferior rectus muscle (IRM) of a smoker (a) and nonsmoker (b). A large high-intensity area (arrow) in the left IRM is illustrated on a T1-weighted coronal magnetic resonance image (a). A small high-intensity area (arrowhead) in the right IRM is demonstrated on a T1-weighted coronal magnetic resonance image (b).

Although there was no significant difference in the largest cross-sectional area of the IRM between smokers and nonsmokers (*P* = 0.887; Table 2), the adipose change was significantly more severe in smokers than in nonsmokers (*P* = 0.010; Figure 2; Table 3). The largest cross-sectional area of the IRM (*P* = 0.347; Table 2) and the degree of adipose change (*P* = 0.549; Table 3) did not differ significantly between patients who had received or had not received previous orbital radiotherapy.

## 4. Discussion

The dose-effect relationship regarding unilateral IRM recession in TED was predicted using IRM thickness, degree of intramuscular adipose change, and smoking status, but not by the previous orbital radiotherapy history.

IRM thickness was found to be positively correlated with the dose-effect relationship for IRM recession. A thick IRM commonly contains a large amount of fibrous tissue [10]. Recession of a fibrotic IRM releases a fibrous contraction [3, 5], resulting in the positive correlation between IRM thickness and the dose-effect relationship.

In contrast, the severity of adipose change showed a negative correlation with the dose-effect relationship regarding IRM recession. Adipose change also results in thickening of the IRM [10] but produces little muscle stiffness and inextensibility. Consequently, ophthalmologists need to estimate the amount of EOM recession on consideration of both IRM thickness and the degree of adipose change in the IRM. MRI can detect both a thickened IRM and adipose change, enabling an accurate preoperative estimation.

Smokers showed a negative correlation (−0.102 of the partial regression coefficient) with the dose-effect relationship for IRM recession. Smokers exhibited a higher degree of adipose change with the same IRM thickness as compared with nonsmokers; that is to say, the amount of fibrous tissue in the IRM was lower in smokers than in nonsmokers, although the volume of the IRM was similar. This resulted in less stiffness and inextensibility of the IRM in smokers, causing the negative correlation. Oxidative stress after smoking disturbs lipoprotein metabolism [14], which may be associated with adipose tissue accumulation in the IRM.

Previous studies have demonstrated no significant difference in the surgical success rate between smokers and nonsmokers [2, 11, 15], which appears to be contrary to the findings of the present study. However, these previous studies simply compared the surgical success rate between smokers and nonsmokers [2, 11, 15], while our study evaluated if there was a correlation between smoking and the dose-effect relationship regarding IRM recession. In addition, the previous studies included all strabismus surgeries, including recession of the horizontal rectus muscles and oblique muscles, resection of the EOMs, bilateral recession, and two muscle recessions on the same side [2, 11, 15]. These previous studies also included patients who had undergone orbital decompression surgery before strabismus surgery [2, 11, 15], which occasionally influences the surgical success of strabismus surgery [16]. These factors may have led to the different results between the present and previous studies.

In the present study, it was found that a history of orbital radiotherapy did not significantly influence the dose-effect relationship regarding IRM recession. This finding may be related to those reported in previous studies; these indicated no significant difference in the surgical success rate between TED patients treated with or without previous orbital radiotherapy, although the inclusion criteria were different [2, 11, 15]. Orbital radiotherapy occasionally leads to orbital fibrosis; however, it decreases TED-related orbital inflammation, which may offset the radiation-induced orbital fibrosis.

The multiple regression model used in the current study was created for unilateral IRM recession. A previous study showed that the dose-effect relationship for EOM recession differed among the EOMs [17]. The present model may, therefore, not be used for recession of the other EOMs. Future studies are necessary to determine other models for recession regarding the other EOMs.

The main methodological limitations of the present study were its retrospective design and small sample size. A larger number of patients would provide a stronger statistical power. Moreover, the use of software to calculate the IRM cross-sectional area would provide more accurate results.

## 5. Conclusions

The IRM thickness, the degree of intramuscular adipose change, and the smoking status, but not the previous history of orbital radiotherapy, were predictors of the dose-effect relationship regarding unilateral IRM recession in TED. Evaluation of the largest IRM cross-sectional area, the degree of adipose change in the IRM, and smoking status will be helpful for estimating the amount of IRM recession.

## Figures and Tables

**Table 1:** Patient data.

Number of patients (male/female)	25 (9/16)
Age (years; range)	61.3 ± 10.1 (38–78)
Surgical side: right/left	13/12
Number of smokers	8
Number of patients with a history of orbital radiotherapy	12

**Table 2:** Inferior rectus muscle thickness in each patient group.

	IRM thickness (mm^2^)
Mean (range)	72.52 ± 17.34 (41.82–117.83)

Smoker (range)	76.51 ± 19.57 (57.23–117.83)
Nonsmoker (range)	70.63 ± 16.49 (41.82–91.30)
*P* value	0.887

Patients with a history of orbital radiation therapy (range)	76.25 ± 20.09 (47.56–117.83)
Patients without a history of orbital irradiation (range)	69.07 ± 14.32 (41.82–91.30)
*P* value	0.347

IRM: inferior rectus muscle.

Statistical comparison using the Mann-Whitney *U* test.

**Table 3:** Relationship between the degree of adipose change in the inferior rectus muscle, smoking status, and history of orbital radiation therapy.

	Adipose change in the IRM				*P* value
Degree	0	1	2	3	

Number of smokers	0	2	2	4	0.010
Number of nonsmokers	6	9	0	2	

Number of patients with a history of orbital radiation therapy	2	7	1	2	0.549
Number of patients without a history of orbital radiation therapy	4	4	1	4	

IRM: inferior rectus muscle.

Statistical comparisons using the chi-square test for independent variables.
